# PI3Kδ Is Essential for Tumor Clearance Mediated by Cytotoxic T Lymphocytes

**DOI:** 10.1371/journal.pone.0040852

**Published:** 2012-07-13

**Authors:** Eva Maria Putz, Michaela Prchal-Murphy, Olivia Annabella Simma, Florian Forster, Xaver Koenig, Hannes Stockinger, Roland P. Piekorz, Michael Freissmuth, Mathias Müller, Veronika Sexl, Eva Zebedin-Brandl

**Affiliations:** 1 Institute of Pharmacology and Toxicology, Department for Biomedical Sciences, University of Veterinary Medicine Vienna, Vienna, Austria; 2 Institute of Animal Breeding and Genetics, Department for Biomedical Sciences, University of Veterinary Medicine Vienna, Vienna, Austria; 3 Immunology Frontier Research Center, Osaka University, Osaka, Japan; 4 Institute for Hygiene and Applied Immunology, Center for Pathophysiology, Infectiology and Immunology, Medical University of Vienna, Vienna, Austria; 5 Department of Neurophysiology and Pharmacology, Center for Physiology and Pharmacology, Medical University of Vienna, Vienna, Austria; 6 Institute of Biochemistry and Molecular Biology II, University Hospital and Clinics, Heinrich-Heine-University, Düsseldorf, Germany; 7 Institute of Pharmacology, Center for Physiology and Pharmacology, Medical University of Vienna, Vienna, Austria; 8 Biomodels Austria, University of Veterinary Medicine Vienna, Vienna, Austria; J. Heyrovsky Institute of Physical Chemistry, Czech Republic

## Abstract

**Background:**

PI3Kδ is a lipid kinase of the phosphoinositide 3-kinase class 1A family and involved in early signaling events of leukocytes regulating proliferation, differentiation and survival. Currently, several inhibitors of PI3Kδ are under investigation for the treatment of hematopoietic malignancies. In contrast to the beneficial effect of inhibiting PI3Kδ in tumor cells, several studies reported the requirement of PI3Kδ for the function of immune cells, such as natural killer and T helper cells. Cytotoxic T lymphocytes (CTLs) are essential for tumor surveillance. The scope of this study is to clarify the potential impact of PI3Kδ inhibition on the function of CTLs with emphasis on tumor surveillance.

**Principal Findings:**

PI3Kδ-deficient mice develop significantly bigger tumors when challenged with MC38 colon adenocarcinoma cells. This defect is accounted for by the fact that PI3Kδ controls the secretory perforin-granzyme pathway as well as the death-receptor pathway of CTL-mediated cytotoxicity, leading to severely diminished cytotoxicity against target cells *in vitro* and *in vivo* in the absence of PI3Kδ expression. PI3Kδ-deficient CTLs express low mRNA levels of important components of the cytotoxic machinery, *e.g. prf1*, *grzmA, grzmB*, *fasl* and *trail*. Accordingly, PI3Kδ-deficient tumor-infiltrating CTLs display a phenotype reminiscent of naïve T cells (CD69^low^CD62L^high^). In addition, electrophysiological capacitance measurements confirmed a fundamental degranulation defect of *PI3Kδ−/−* CTLs.

**Conclusion:**

Our results demonstrate that CTL-mediated tumor surveillance is severely impaired in the absence of PI3Kδ and predict that impaired immunosurveillance may limit the effectiveness of PI3Kδ inhibitors in long-term treatment.

## Introduction

The common catalytic function of phosphoinositide 3-kinases (PI3Ks) is the phosphorylation of the D3-position of phosphatidylinositol. The PI3K family consists of three classes based on their primary structure, regulation, and *in vitro* liquid substrate specificity. Class I PI3Ks catalyze the phosphorylation of phosphatidylinositol 4,5-bisphosphate (PIP_2_) and thereby generate phosphatidylinositol 3,4,5-triphosphate (PIP_3_). PIP_3_ is selectively recognized by some pleckstrin homology domains and thus provides a membrane docking site for many different proteins, *e.g.* the serine-threonine-kinase AKT and its upstream activator the phosphoinositide-dependent kinase-1 (PDK1), the guanine nucleotide exchange factors for ARF6 ARNO (ARF nucleotide-site opener), the general receptor of phosphoinositide-1 (GRP1), and non-receptor tyrosine kinases of the BTK and TEC-family. Accordingly, class I PI3Ks impinge on many cellular signaling cascades, which affect cell growth and survival, trafficking of vesicles and dynamics of the actin cytoskeleton. As a consequence, the PI3K/AKT/mTOR pathway has been shown to play an important role in apoptosis and cancer [1].

Class I PI3Ks are heterodimeric molecules comprising a catalytic and a regulatory subunit. There are four catalytic isoforms of class I PI3Ks (class IA p110α, p110β, p110δ and class IB p110γ). The isoforms p110α and p110β are ubiquitously expressed, whereas p110δ and p110γ are predominantly expressed in the hematopoietic system [2], [3]. Currently, tools to study PI3K signaling range from genetically modified mouse strains either lacking individual class I PI3K isoforms or harboring point mutations giving rise to catalytically inactive proteins, to PI3K isoform-specific small-molecule inhibitors [4].

T lymphocytes are of particular interest because they express all four catalytic isoforms. The enzymes can therefore be envisaged to have both, redundant and unique functions. In fact, T cells develop normally in mice with engineered deletions or kinase-dead (KD) versions of PI3Kδ [5], [6], but in PI3Kγ-deficient mice T cells show partial defects in β-selection [7]. In contrast, mice deficient in both, PI3Kδ and PI3Kγ, suffer from a profound block at the pre-T cell receptor (pre-TCR) selection step of thymus development. In these mice the numbers of splenic CD4+ and CD8+ T cells are significantly reduced and the majority of peripheral CD4+ T cells display a memory phenotype [8], [9]. Using small-molecule inhibitors, Ji et al [8] demonstrated that in mature T cells PI3Kδ, but not PI3Kγ, controls Th1 and Th2 cytokine secretion.

PI3Kδ is a key component of the signaling machinery downstream of the TCR and CD28 [10] and it is the most relevant isoform responsible for PIP_3_ accumulation at the immunological synapse upon TCR activation [10], [11]. Hence, PI3Kδ-deficient CD4+ T helper (Th) cells display defects in antigen-presenting cell-mediated stimulation and clonal expansion *in vivo* and *in vitro* [12]. PI3Kδ-KD CD4+ T cells proliferate moderately slower in response to anti-CD3 (aCD3) stimulation, but this defect is rescued by antibody-dependent co-stimulation of CD28 [10]. Upon physiological stimuli PI3Kδ-KD Th cells show reduced differentiation along the Th1 and Th2 lineages [12]. As a consequence of reduced Th2 responses, PI3Kδ-deficient mice are protected from experimentally-induced airway inflammation [13]. Additionally, a study by Haylock-Jacobs et al [14] showed that PI3Kδ is a key player in the pathophysiology of experimental autoimmune encephalomyelitis (EAE), a Th17-driven model of multiple sclerosis. Furthermore, loss of PI3Kδ was also shown to compromise the function of regulatory T cells [15], another CD4+ T cell lineage.

While the role of PI3Kδ in CD4+ T cells is understood in considerable detail, it is not clear whether the enzyme is important for the function of CD8+ cytotoxic T lymphocytes (CTLs). Thus, the aim of the present study was to determine the consequences of PI3Kδ-deficiency on CTL functions *in vitro* and *in vivo.* Our observations clearly show that PI3Kδ is indispensable at several stages of CTL biology. PI3Kδ-deficiency impedes the activation of CTLs and gives rise to inactive and quiescent CTLs, whose composition of the lytic machinery required for degranulation and target cell lysis is altered and functionally impaired. This defect severely curtails CTL-mediated antigen-specific cytotoxicity and impairs tumor surveillance. PI3Kδ-deficient mice develop significantly bigger solid tumors after inoculation with MC38 colon carcinoma cells. These results evoke safety concerns and challenge the use of PI3Kδ inhibitors in cancer treatment. Impaired CTL-mediated immunosurveillance might limit the effectiveness of PI3Kδ inhibitors by counteracting intended treatment effects on tumor target cells. However, we suggest that PI3Kδ inhibitors might be of therapeutic relevance in areas where suppression of CD8+ T cells is useful, *e.g.* in transplantation medicine or in the treatment of autoimmune diseases and chronic obstructive pulmonary disease (COPD).

## Results

### PI3Kδ is Required to Induce T Cell Responses to Allogeneic Lymphocytes

As PI3K p110 isoforms might have redundant functions, we determined expression levels of PI3Kα, β, and γ in *PI3Kδ−/−* CTLs. **Figure S1** illustrates no major alterations or compensations of other PI3K p110 isoforms in *PI3Kδ−/−* CTLs.

**Figure 1:**
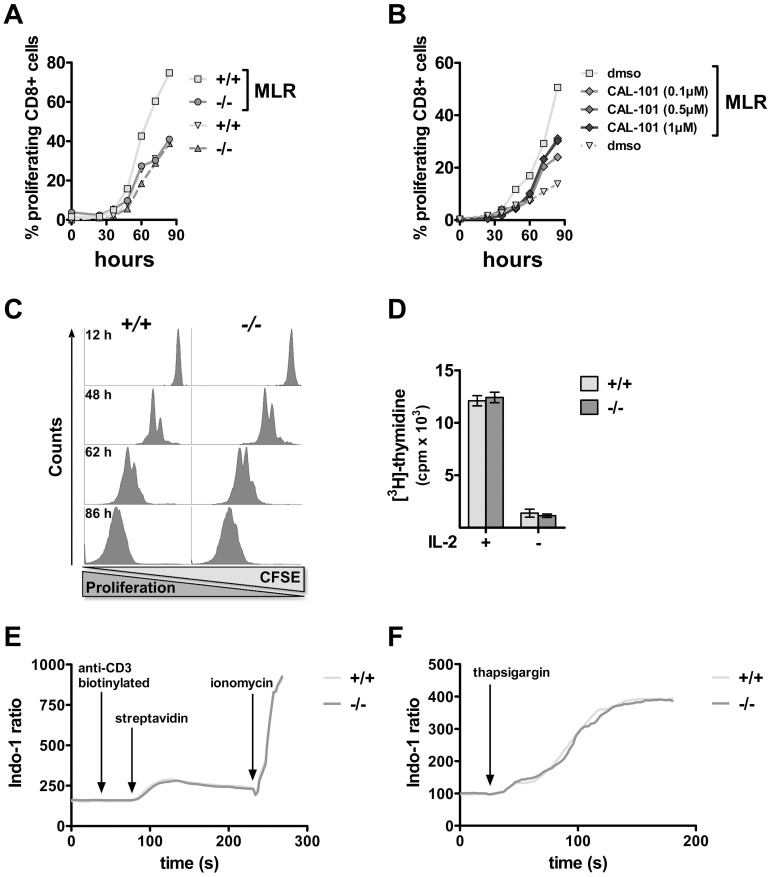
Diminished reaction of *PI3Kδ−/−* CTLs to allogeneic mixed lymphocytes, but unaltered proliferation and Ca^2+^-response. A. WT and *PI3Kδ−/−* splenocytes were CFSE-labeled and cultured in the absence and presence of allogeneic (BALB/c), mitomycin C-treated splenocytes. At the indicated time points, cells were harvested and proliferation of responding CTLs was assessed by flow cytometry. Percentages of proliferating CFSE+CD8+ T cells with and without the stimulus of mixed lymphocytes are illustrated. Proliferating CD8+ T cells were discriminated from undivided T cells by the reduced levels of CFSE in daughter cells. B. WT splenocytes were CFSE-labeled and cultivated in analogy to A. Pharmacological inhibition of PI3Kδ was achieved by treatment with indicated concentrations of CAL-101 during the experimental procedure. DMSO-treatment served as negative control. C. Proliferation of WT and *PI3Kδ−/−* CTLs in response to aCD3ε treatment was assessed in a CFSE proliferation assay. D. Proliferation of WT and *PI3Kδ−/−* aCD3-activated T cells was assessed under standard T cell medium conditions (in the presence of IL-2) and after deprivation from IL-2 by performing an [^3^H]-thymidine incorporation assay over 48 hours (with IL-2: WT: 12097±491cpm; versus *PI3Kδ−/−*: 12413±501cpm; without IL-2: WT: 1392±381cpm; versus *PI3Kδ−/−*: 1140±160cpm, n = 6, values represent mean±SD). E., F. WT and *PI3Kδ−/−* splenocytes were stained with 1 µM Indo-1 AM. Ca^2+^ flux in response to aCD3ε followed by crosslinking with streptavidin (E) or thapsigargin (F) was measured in CD8+ T cells using flow cytometry. Treatment with ionomycin served as positive control. Three independent experiments were carried out and one representative experiment is shown, respectively.

The mixed lymphocyte reaction (MLR) shall induce T cell activation and enhanced proliferation. We challenged carboxyfluorescein succinimidyl ester (CFSE)-labeled wild type and *PI3Kδ−/−* splenocytes (C57BL/6 background) *in vitro* with allogeneic lymphocytes from BALB/c mice (different MHC haplotype). Over a period of 84 hours the proliferation of CD8+ CFSE-labeled cells was assessed *via* flow cytometry according to the incremental reduction in CFSE-intensity due to cell division (**Figure 1A**). *PI3Kδ−/−* CTLs failed to react with enhanced proliferation upon challenge with allogeneic antigens, as their growth rates were comparable to unstimulated CD8+ T cells. Additionally, we performed pharmacological inhibition of PI3Kδ with CAL-101. This potent and selective inhibitor of PI3Kδ has already paved its way into human clinical trials [16], for review see [17], [18]. The efficient inhibition of PI3Kδ by CAL-101 was confirmed by abrogated phosphorylation of the downstream target AKT. Further, we ruled out that *in vitro* treatment of CTLs with CAL-101 evoked toxic effects (**Figure S2**). Pharmacological inhibition of PI3Kδ resulted in a reduced proliferative response of CD8+ CFSE-labeled cells towards allogeneic lymphocytes, mimicking the phenotype of *PI3Kδ−/−* CTLs **(** **Figure 1B**). In contrast, wild type CTLs were rapidly activated and the cells proliferated significantly faster upon co-incubation with allogeneic splenocytes. These differences were not related to a general inability of *PI3Kδ−/−* CTLs to divide and grow, as proliferation in response to anti-CD3ε (**Figure 1C**) and interleukin-2 (IL-2, **Figure 1D**) was unaltered. Similarly, we failed to detect any alterations in the apoptotic behavior of *PI3Kδ−/−* CTLs after IL-2 withdrawal: apoptosis rates in wild type and *PI3Kδ−/−* CTLs were comparable, which was assessed via cell cycle staining with propidium iodide (data not shown). These experiments led us to the conclusion that PI3Kδ is dispensable for CTL proliferation *per se*, but required in the activation process of CD8+ CTLs when challenged with allogeneic lymphocytes.

Calcium (Ca^2+^) is released in response to TCR activation and is the final step to trigger CTL response. It was shown previously that Ca^2+^ influx into *PI3Kδ−/−* CD4+ T cells was impaired [19]. Accordingly, we examined, whether a defect in early TCR signaling or altered Ca^2+^- concentration in intracellular stores accounted for the missing response of *PI3Kδ−/−* CTLs to foreign antigens. Intracellular Ca^2+^-flux was monitored after preloading cells with the fluorescent indicator dye Indo-1. Importantly, PI3Kδ-deficiency did not interfere with Ca^2+^-influx triggered by TCR-crosslinking, indicating that early TCR signaling is intact in *PI3Kδ−/−* CD8+ T cells (**Figure 1E**). We also analyzed the size of the intracellular releasable storage pool in the endoplasmic reticulum by blocking the sarco/endoplasmic reticulum Ca^2+^-ATPase with thapsigargin. The ensuing increase in intracellular Ca^2+^ was virtually identical in *PI3Kδ−/−* and wild type CTLs (**Figure 1F**). These experiments ruled out that altered Ca^2+^-mobilization accounted for any functional failure of PI3Kδ-deficient CTLs.

### PI3Kδ is Needed to Arm CTLs

**Figure 2:**
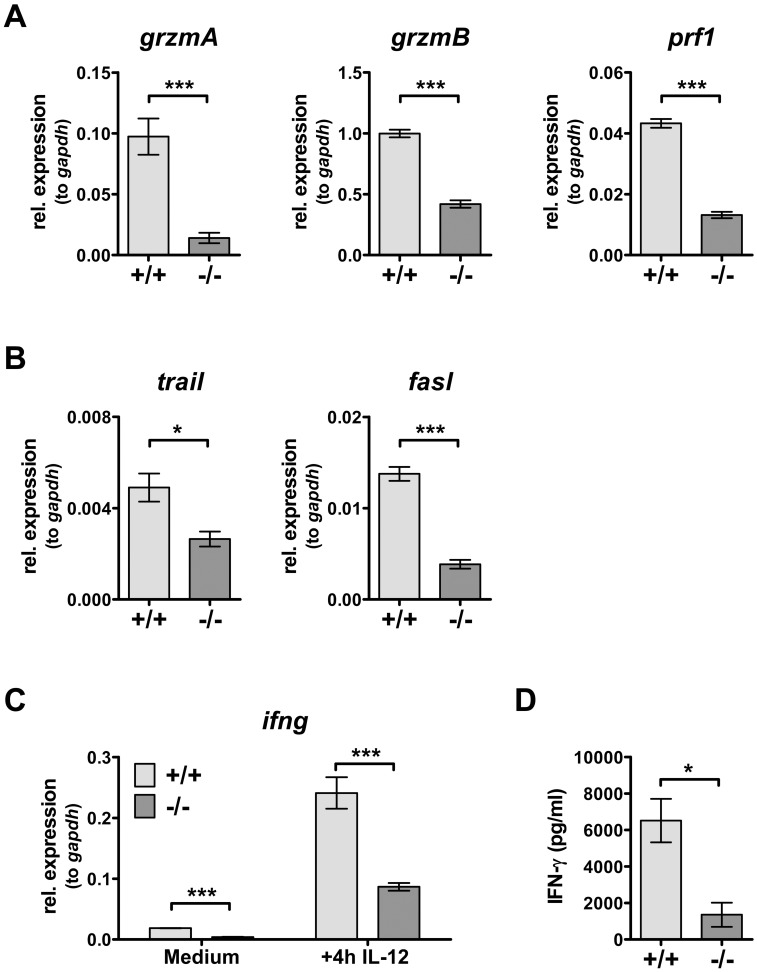
Reduced expression of cytotoxic components in *PI3Kδ−/−* CTLs. WT and *PI3Kδ−/−* splenocytes were activated for 3 days with aCD3ε and cultured in T cell medium. A. mRNA expression of *grzmA* (WT: 0.097±0.036; versus *PI3Kδ−/−*: 0.014±0.01, n = 6, *p = 0.0003*), g*rzmB* (WT: 1±0.076; versus *PI3Kδ−/−*: 0.419±0.075, n = 6, *p<0.0001*) and *prf1* (WT: 0.0433±0.004; versus *PI3Kδ−/−*: 0.013±0.003, n = 6, *p<0.0001*) was quantified by qRT-PCR and normalized to the house-keeping gene g*apdh*. B. Similarly, under standard culturing conditions mRNA levels of *trail* (WT: 0.0049±0.0012; versus *PI3Kδ−/−*: 0.0026±0.0007, n = 4, *p = 0.0178*) and *fasl* (WT: 0.0138±0.0015; versus *PI3Kδ−/−*: 0.0039±0.0001, n = 4, *p<0.0001*) were measured. C. *Ifng* mRNA was quantified by qRT-PCR under standard culturing conditions (WT: 0.019±0.0005; versus *PI3Kδ−/−*: 0.004±0.001, n = 6, *p<0.0001*) and after stimulation with 5 ng/ml IL-12 for 4 h (WT: 0.241±0.06; versus *PI3Kδ−/−*: 0.087±0.016, n = 6, *p = 0.0002*). D. To quantify IFN-γ protein levels, WT and *PI3Kδ−/−* splenocytes were stimulated with ConA. After 48 h supernatants were harvested and IFN-γ release was measured by ELISA (WT: 6515±2061 pg/ml; versus *PI3Kδ−/−*: 1359±1147 pg/ml, n = 3, *p = 0.0193*). IFN-γ release of unstimulated controls of WT and *PI3Kδ−/−* splenocytes was below detection limit of the assay (<10 pg/ml). Statistics were calculated with an unpaired Student’s *t*-test, and values represent mean±SD. One out of two independently performed experiments with comparable results is shown.

The major task of CTLs is the eradication of cells containing non-self-antigens, *i.e.* cancer cells or virally infected cells. For their cytotoxic action CTLs rely on lytic granules filled with proteolytic enzymes such as granzymes and perforin. We evaluated the expression of these components in aCD3-activated CTLs and found significantly reduced mRNA levels for g*rzmA*, *grzmB* and *prf1* in *PI3Kδ−/−* CTLs (**Figure 2A**). This indicates that PI3Kδ is required to arm CTLs for efficient lysis. Moreover, CTLs are equipped with the death receptors TNF-related apoptosis-inducing ligand (TRAIL) and Fas ligand (FasL), which can directly induce apoptosis in target cells upon linkage to the respective counterparts. As shown in **Figure 2B**, PI3Kδ-deficient CTLs express significantly lower mRNA levels of *trail* and *fasl*. Apart from granule-exocytosis and the death-receptor cytotoxicity pathway, activated CTLs produce prominent amounts of interferon-γ (IFN-γ) which supports the antiviral response of the host. Again, also *ifng* mRNA was substantially reduced in the absence of PI3Kδ (**Figure 2C**). Accordingly, even when stimulated with the plant lectin concanavalin A for 48 h, *PI3Kδ−/−* splenocytes released less IFN-γ protein than wild type splenocytes (**Figure 2D**).

### Granule Release by CTLs Depends on PI3Kδ

**Figure 3:**
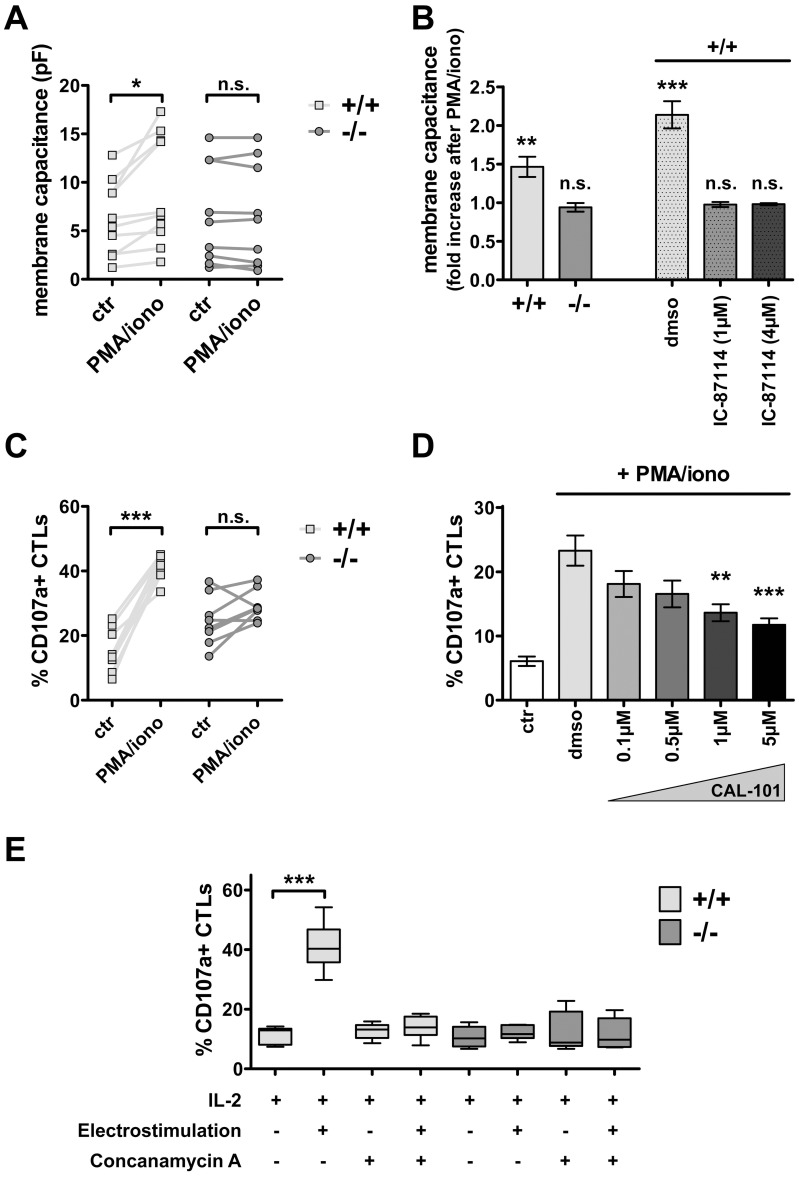
PI3Kδ is indispensable for CTL degranulation. A. Using the whole cell patch clamp technique, the cellular capacitance of aCD3-activated and in T cell medium cultivated WT and *PI3Kδ−/−* CTLs was determined. Membrane capacitances before (ctr) and after stimulation with PMA and ionomycin (PMA/iono) at the single cell level are summarized from two representative experiments (WT: ctr: 6.3±1.2pF, PMA/iono: 9±1.8pF, n = 10, *p = 0.01*; versus *PI3Kδ−/−*: ctr: 6.7±1.7pF, PMA/iono: 6.6±1.8, n = 9, *p = 0.4*; paired *t*-test; values represent mean±SEM). B. Fold increase in membrane capacitance due to stimulation with PMA/iono is calculated (WT: 1.46±0.13, n = 10, *p = 0.0064*; versus *PI3Kδ−/−*: 0.94±0.06%, n = 9, *p = 0.3*). Additionally, WT CTLs were pre-incubated with the PI3Kδ isoform-specific inhibitor IC-87114 (1 µM, 4 µM, 1 hour), DMSO-treatment served as control (DMSO: 2.14±0.18, n = 11, *p<0.0001*; 1 µM IC-87114: 0.98±0.03, n = 11, *p = 0.49*; 4 µM IC-87114: 0.98±0.01, n = 9, *p = 0.18*; values representing mean±SEM, One sample *t*-test). C. *In vitro* cultivated WT and *PI3Kδ−/−* CTLs were challenged with PMA and ionomycin. The percentage of CD107a+ T cells was measured *via* flow cytometry before and after the stimulus (WT: ctr: 15.5±2.4%, PMA/iono: 41.2±1.3%, *p<0.0001*; versus *PI3Kδ−/−*: ctr: 24.6±2.7%, PMA/iono: 29.3±1.7%, *p = 0.085*; n = 8, paired *t*-test, values represent mean±SEM). D. Accordingly, the degranulation of WT CTLs upon pharmacological inhibition of PI3Kδ using CAL-101 was tested (ctr: 6±0.7%; +PMA/iono: DMSO: 23.3±2.4%, 0.1 µM: 18.1±2%, 0.5 µM: 16.6±2.1%, 1 µM: 13.6±1.3%, 5 µM: 11.7±1%, n≥6, values represent mean±SEM. One-Way ANOVA revealed p<0.0001, Tukey’s Post-Hoc test was significant *p<0.01* for 1 µM and highly significant *p<0.001* for 5 µM compared to DMSO control). E. WT and *PI3Kδ−/−* CTLs were cultivated in T cell medium and their expression of CD107a was measured *via* flow cytometry under basal conditions and after electrostimulation or electrostimulation plus the degranulation inhibitor Concanamycin A, respectively (WT: ctr: 11.5±1.2% CD107a+ cells, electrostimulation: 41±3.3% CD107a+ cells, *p = 0.0001*; versus *PI3Kδ−/−*: ctr: 11±1.3% CD107a+ cells, electrostimulation: 12±0.8% CD107a+ cells, *p = 0.54*, paired *t*-test, values represent mean±SEM). All experiments were performed at least two times independently and summarized in the depicted graphs.

So far, our data indicate that PI3Kδ is required to equip the lytic granules of CTLs with cytotoxic components. We used two approaches to test whether PI3Kδ is involved in the degranulation process. The first approach was performed on the single cell level and relied on the recording of cellular membrane capacitance by electrophysiological measurements. Upon degranulation lytic granules fuse with the cell membrane and lead to an increase in cellular surface area, which corresponds to a quantifiable rise in membrane capacitance. This alteration in membrane capacitance can be recorded with the whole cell patch clamp technique at the single cell level. Degranulation was induced by providing activating signals, *i.e.* stimulation of protein kinase C isoforms by the phorbol ester PMA and calcium influx by the calcium ionophore ionomycin. **Figure 3A** summarizes the membrane capacitance recordings of wild type and *PI3Kδ−/−* CTLs under basal conditions and after superfusion with PMA and ionomycin. Corresponding to differences in cell size, wild type and *PI3Kδ−/−* CTLs displayed a distinct range of basal membrane capacitances. However there was no significant difference in basal cellular capacitance between wild type and *PI3Kδ−/−* CTLs. Stimulation of wild type cells triggered a significant increase of approximately 1.5 fold cell capacitance, independent of their basal cell size (**Figure 3B**). In contrast, no significant increase in cellular capacitance was detectable after superfusion of *PI3Kδ−/−* CTLs. Accordingly, upon pharmacological inhibition of PI3Kδ in wild type cells no significant increase in cellular capacitance could be induced, as compared to untreated DMSO controls.

The second approach was based on flow cytometry and allowed for the analysis of a large cell population: the lysosomal marker CD107a is expressed on the cell surface at significant levels only after degranulation and thus has been used as a surrogate marker of cytotoxicity [20]. We therefore analyzed CD107a levels by flow cytometry under basal conditions and after stimulation with PMA and ionomycin. This combination elicited a prominent increase in surface expression of CD107a in wild type cells. In contrast, CD107a expression was not significantly increased upon PMA/ionomycin stimulation of *PI3Kδ−/−* CTLs (**Figure 3C** **)**. Additionally, the impact of pharmacological inhibition of PI3Kδ in wild type CTLs was investigated. The induction of degranulation was significantly decreasing proportional to the applied CAL-101 concentrations as compared to DMSO-treated cells (**Figure 3D**). Treating cells with pharmacological compounds may interfere with more than one reaction or induce off-target effects, *e.g.* phorbol esters bind to MUNC13 (mammalian homologous of the C-elegans unc-13 gene), a component of the vesicle fusion machinery [21]. To confirm our data we also employed an independent stimulus to trigger degranulation again *via* elevation of intracellular Ca^2+^: electrical field stimulation (chronic low frequency stimulation, CLFS, 1 Hz). As electrical field stimulation has not been used in this context before, we employed several control experiments to prove the specific nature of the stimulatory effect. No significant increase in CD107a expression was induced, when Ca^2+^ was removed from culture media (data not shown). Importantly, *PI3Kδ−/−* CTLs did not display any detectable degranulation upon electrostimulation, whereas wild type CTLs expressed high levels of CD107a on their surface (**Figure 3E**). When CTLs were pre-treated with 10 nM concanamycin A, an inhibitor of perforin-mediated cytotoxicity [22], both CLFES (**Figure 3E**) and pharmacological stimulation (not shown) did not lead to increased CD107a-surface expression. To sum up, both, the single cell- as well as the population-based approach verified that fusion of lytic granules with the cellular membrane was impaired in *PI3Kδ−/−* CTLs or due to pharmacological inhibition of PI3Kδ.

### PI3Kδ-deficiency Impairs Antigen-specific Response *in vitro* and cytotoxicity of CTLs *in vivo*

**Figure 4:**
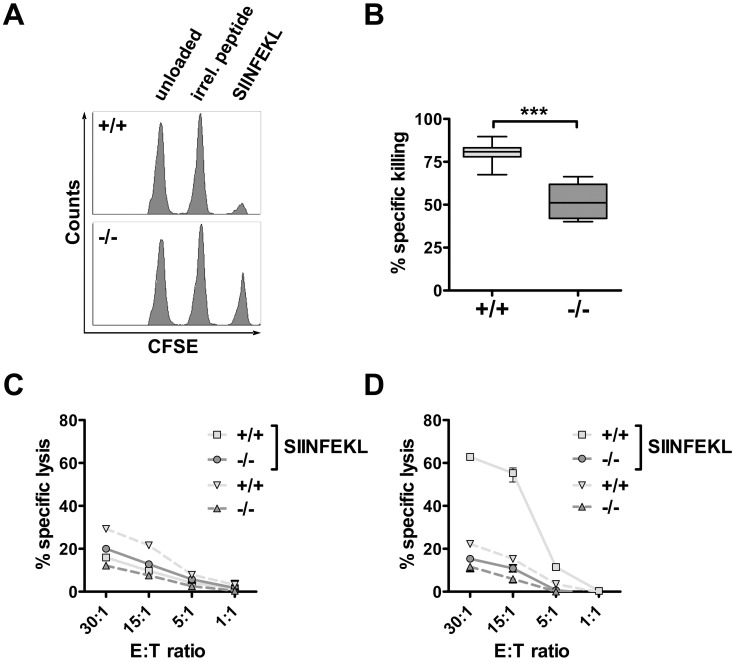
PI3Kδ-deficiency leads to severely impaired CTL cytotoxicity *in vivo* and *in vitro*. A., B. WT and *PI3Kδ−/−* animals were immunized with SIINFEKL and CpG. Seven days later mice received CFSE-labeled targets and peptide-specific CTL activity in draining inguinal lymph nodes was analyzed by flow cytometry. A. A representative FACS histogram for each genotype is depicted. B. SIINFEKL-specific target cell killing was calculated from three independent experiments as described in *Materials and Methods* (WT: 80±6% antigen-specific lysis; versus *PI3Kδ−/−*: 52±10% antigen-specific lysis, n = 12; values represent mean±SD, unpaired *t*-test, *p<0.0001*). No specific killing was observed in control mice (data not shown). C., D. WT and *PI3Kδ−/−* animals were immunized with SIINFEKL peptide and CpG and boosted 7 days later. To generate effector cells, splenocytes were isolated at day 14 and co-cultured for 5 days with irradiated SIINFEKL-pulsed splenocytes. To determine peptide-reactive CTL cytotoxicity *in vitro*, CFSE-labeled EL4 (C) and OVA-expressing EG7 (D) target cells were co-cultured with effectors in ratios of 30∶1, 15∶1, 5∶1 and 1∶1. Specific *in vitro* target cell killing was quantified by flow cytometry (EG7, E:T = 30∶1: WT: 63% specific lysis; versus *PI3Kδ−/−*: 15% specific lysis). One representative experiment out of three is shown.

So far we demonstrated that *PI3Kδ−/−* CTLs do not respond to foreign antigens with enhanced proliferation, that they express reduced levels of important components of the lytic machinery, and that they have a severe degranulation defect. Hence, all these data reveal an essential role of PI3Kδ for CTL-mediated cytotoxicity at different stages in the canonical killing pathway. To investigate the antigen-specific cytotoxicity of CTLs *in vivo,* wild type and *PI3Kδ−/−* mice were immunized subcutaneously with the SIINFEKL peptide in combination with an adjuvant (CpG). One week thereafter mice received syngeneic target cells containing CFSE^low^ splenocytes, CFSE^mid^ splenocytes pulsed with an irrelevant peptide and CFSE^high^ splenocytes pulsed with SIINFEKL mixed in a 1∶1∶1 ratio intravenously. Following 18 hours, the draining inguinal lymph nodes and spleens of wild type and *PI3Kδ−/−* mice were analyzed by flow cytometry. **Figure 4A** depicts a representative histogram plot obtained from lymph nodes of immunized wild type and *PI3Kδ−/−* mice: whereas the splenocytes loaded with SIINFEKL peptide were drastically reduced in lymph nodes from wild type mice after immunization, this effect was observed to a significantly lower extent in *PI3Kδ−/−* mice (summarized in **Figure 4B**). These experiments document a reduced cytotoxic ability of CTLs in the absence of PI3Kδ. To further support this finding, we additionally challenged *PI3Kδ−/−* CTLs in an *in vitro* experimental set-up. We again immunized wild type and *PI3Kδ−/−* mice with SIINFEKL peptide plus adjuvant over a period of two weeks. Thereafter, splenocytes were prepared and co-cultivated with SIINFEKL-loaded splenocytes for five days. The thymoma cell lines EL4 and its corresponding ovalbumin (OVA)-expressing cell line EG7 were used to test the cytolytic capacity of the splenocytes. EL4 cells should not be recognized and were used as targets to determine unspecific background killing, which was comparable between wild type and *PI3Kδ−/−* CTLs (**Figure 4C**). In contrast, OVA-expressing EG7 cells were efficiently recognized and killed by primed wild type CTLs, whereas primed *PI3Kδ−/−* CTLs failed to induce any detectable antigen-specific cytotoxicity above background levels (**Figure 4D**). To confirm the impact of PI3Kδ on CTL-mediated antigen-specific cytotoxicity we isolated splenocytes from OT-I mice and co-cultivated them with SIINFEKL-loaded splenocytes as described above. The CTLs were treated with the PI3Kδ inhibitor CAL-101 2 hours prior and during the *in vitro* cytotoxicity assay. Again, OVA-expressing EG7 cells were efficiently lysed by DMSO-treated OT-I CTLs, whereas CAL-101-treated OT-I cells showed significantly reduced cytotoxicity (**Figure S3**).

### PI3Kδ is Required for CTL Activation and Tumor Surveillance

**Figure 5:**
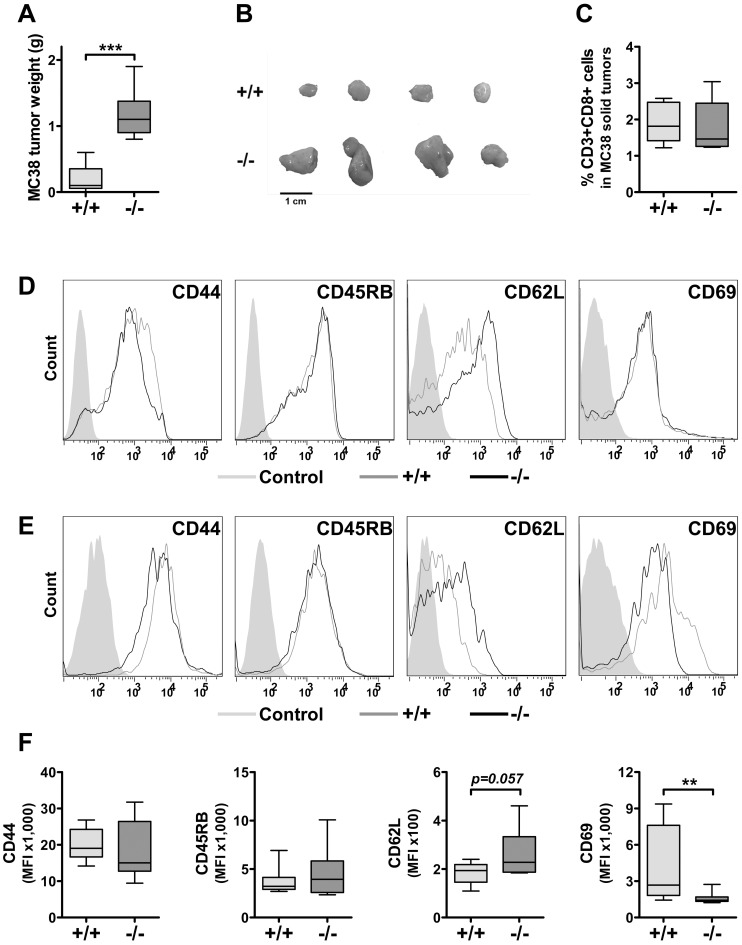
Impaired activation of *PI3Kδ−/−* CTLs by tumor cells. A. 1×10^6^ MC38 colon adenocarcinoma cells were injected subcutaneously into the flanks of WT and *PI3Kδ−/−* animals. Fifteen days later tumor weights were analyzed. Depicted is the summary of 3 independent experiments (WT: 0.19±0.19 g; versus *PI3Kδ−/−*: 1.16±0.33 g, n = 12, values represent mean±SD). B. Representative pictures of MC38 solid tumors grown in WT and *PI3Kδ−/−* animals. C. After removal, tumors were minced and digested with collagenase D and DNase I. Tumor-infiltrating CD3+CD8+ CTLs were quantified using flow cytometry (WT: 1.9±0.5%; versus *PI3Kδ−/−*: 1.8±0.7%, n = 8, values represent mean±SD). D., E. Representative histograms showing expression levels of CD44, CD45RB, CD62L and CD69 on splenic CD3+CD8+ T cells from untreated WT and *PI3Kδ−/−* mice (D) and on MC38 tumor-infiltrating CD3+CD8+ CTLs of WT and *PI3Kδ−/−* mice (E). F. Summary of the expression levels of CD44 (WT: median = 19040, IQR = 16670–24220; versus *PI3Kδ−/−*: median = 15053, IQR = 12750–26440, n = 8), CD45RB (WT: median = 3222, IQR = 2907–4142; versus *PI3Kδ−/−*: median = 3945, IQR = 2579–5833, n = 8), CD62L (WT: median = 194, IQR = 146–219; versus *PI3Kδ−/−*: median = 228, IQR = 187–333, n = 8, Mann-Whitney test: *p = 0.0572*) and CD69 (WT: median = 2673, IQR = 1825–7606; versus *PI3Kδ−/−*: median = 1428, IQR = 1334–1698, n = 8, Mann-Whitney test: *p = 0.0037*) on tumor-infiltrating WT and *PI3Kδ−/−* CD3+CD8+ CTLs.

At this point we speculated that PI3Kδ inhibition – besides beneficial effects on tumor growth restriction – might entail adverse effects on the immune system. Thus, we wanted to challenge the concept of PI3Kδ inhibition in the treatment of malignancies and tested whether impaired CTL-mediated cytotoxicity observed in *PI3Kδ−/−* mice was indeed relevant for tumor surveillance. We made use of the colon adenocarcinoma cell line MC38, which is recognized and lysed in a CTL-dependent manner [23]. Fifteen days after subcutaneous injection of 10^6^ cells into the flanks of wild type and *PI3Kδ−/−* mice, the animals were sacrificed and the tumors analyzed. As depicted and summarized in **Figure 5A and 5B** significantly larger tumors had developed in *PI3Kδ−/−* mice compared to wild type recipients. When we characterized the tumor-infiltrating lymphocytes, we found comparable numbers of CD3+CD8+ effector cells irrespective whether a tumor evolved in wild type or *PI3Kδ−/−* recipients (**Figure 5C**). Investigating resting CTLs from untreated healthy mice, we found comparable expression profiles of the activation markers CD45RB and CD69 on wild type and *PI3Kδ−/−* lymphocytes, whereas the expression of CD44 was reduced in *PI3Kδ−/−* lymphocytes (**Figure 5D**). Pharmacological inhibition of PI3Kδ in mature wild type CTLs over a time period of 3 days resulted in reduced expression of CD44 while no difference was observed in the expression of CD69 compared to DMSO-treated wild type controls (**Figure S4**). On tumor-infiltrating CTLs the expression of CD44 and CD45RB was comparable (**Figure 5E, F**); no consistent and significant differences in their expression levels could be detected. In contrast, we found a significantly reduced expression of the activation marker CD69 on *PI3Kδ−/−* tumor-infiltrating CTLs. Thus, although *PI3Kδ−/−* CTLs migrated to the tumor, they were activated to a lower extent. Further proof for this concept was obtained by analyzing CD62L, which is down-regulated upon activation. CD62L was consistently higher in *PI3Kδ−/−* CTLs (compare **Figure 5D** depicting expression profiles of unchallenged, resting CTLs and **Figure 5E, F** showing expression levels on tumor-infiltrating CTLs). Moreover, prolonged pharmacological inhibition of PI3Kδ in splenic wild type CTLs resulted in significantly increased expression of CD62L compared to DMSO-treated wild type controls (**Figure S4**). Splenic CD8+ T cells comprise different subsets, amongst which naïve T cells are the most prevalent ones. We could not observe any toxic effect of CAL-101 on the entity of CD8+ T cells. Hence, we are convinced that the differences in CD44 and CD62L are not the result of different CD8+ subset susceptibilities to CAL-101.

In summary, these experiments clearly verified reduced CTL-mediated tumor surveillance in *PI3Kδ−/−* mice accompanied by decreased activation of *PI3Kδ−/−* CTLs (**Figure 5D-F**).

## Discussion

T cells express all four catalytic isoforms of PI3K. Robust networks are engineered to be resilient by built-in redundancies: because of redundant trajectories, removal of one nod does not affect the flow of signals through the network. In each instance, the product of PI3Ks’ enzymatic catalysis is PIP_3_, a lipid second messenger that supposedly activates identical effectors. It was therefore surprising to observe that removal of PI3Kδ had such a profound effect on the function of CD8+ cytotoxic cells supported by the following key findings: (i) The components required for their eponymous action were only expressed at low levels in PI3Kδ-deficient CTLs (granzyme A and B, perforin, FasL, TRAIL, and IFN-γ). (ii) Granule release and (iii) antigen-induced clonal expansion was impaired in PI3Kδ-deficient CTLs. (iv) Predictably, in the absence of PI3Kδ, CTLs failed to restrict MC38 tumor cell growth *in vivo*. Our findings suggest that the absence of PI3Kδ in CD8+ T cells phenocopies the deficiencies that have been observed in PDK1-deficient CTLs and CTLs treated with an inhibitor of AKT1 [24], suggesting that low expression levels of granzymes, perforin and FasL might be due to disrupted AKT-dependent phosphorylation of Foxo3 (forkhead box O3). Similarly, CTLs treated with an AKT1-inhibitor or deficient in PDK1 also failed to mount a proliferative response upon antigen challenge. PIP_3_, the second messenger produced by PI3Ks, activates clearly more effectors than only AKT and PDK1. However, PI3Kδ-deficiency, AKT-inhibition and abrogation of PDK1 expression result all in similar phenotypic consequences, *i.e.* impaired expression of cytotoxic proteins. Hence we speculate that the pertinent signaling module that mediates extracellular input to transcriptional control is composed of PI3Kδ, PDK1, AKT and Foxo3. In this module, the requirement for PI3Kδ is absolute; the other PI3K-isoforms cannot compensate for its absence. Similarly, antigen-induced, TCR-dependent cell proliferation is contingent on the module comprising PI3Kδ, PDK1 and AKT. Our observations suggest that this is not necessarily the case for other stimuli such as IL-2, a conclusion that was reached by McIntyre et al [24] in a similar way.

CTLs can kill infected or malignant cells *via* degranulation. We demonstrate here for the first time that PI3Kδ is indispensable for degranulation of CTLs. Our observations extend comparable findings with mast cells [25] and Natural Killer (NK) cells [26]. Whereas the indispensable role of PI3Kδ in degranulation and cytokine secretion is beyond dispute, its relevance for NK cell cytotoxicity is seen controversially [26]–[31]. It is very likely that the deficiency in PI3Kδ impairs degranulation by effector mechanisms other than downstream signaling *via* AKT. The following arguments support this conjecture: (i) Capacitance measurements study the very last step of the fusion reaction at the single cell level, *i.e.* the calcium-dependent incorporation of vesicular membrane into the cell surface. Electrophysiological recordings allow for unrivaled time resolution in the sub-millisecond range. The kinetics of the capacitance change are not compatible with an intervening enzymatic cascade [26], [32]. The absence of PI3Kδ impaired this very rapid fusion event, suggesting that the depletion of PIP_3_ interferes with the fusion *per se*. (ii) Electrical field stimulation triggers the fusion event directly, *i.e.* by calcium influx and presumably by calcium-dependent activation of SNARE proteins (soluble NSF-attachment receptors) [33]. PI3Kδ-deficient CTLs failed to exocytose and to expose CD107a despite unaltered calcium responses. (iii) Phosphoinositides are known to be asymmetrically distributed. Due to their acidic nature they have been proposed to contribute to SNARE-dependent fusion [34]. In addition, Low et al [35] demonstrated a contribution of PI3Kδ to membrane trafficking at the trans-Golgi network. This resulted in impaired cytokine-secretion in macrophages and might also contribute to the reduced secretion of IFN-γ of PI3Kδ-deficient CTLs that we observed.

The disclosure of the indispensable role of PI3Kδ for CTL function led us to envision potential limitations or safety concerns regarding the clinical applicability of PI3Kδ inhibitors in humans. Thus, we explored how PI3Kδ-deficiency affected CTL-mediated tumor surveillance *in vivo*, thereby challenging the therapeutic concept of PI3Kδ inhibition. *PI3Kδ−/−* CTLs infiltrated evolving tumors with unaltered frequency, but CD69 expression was severely reduced. Increase in CD69 expression is directly linked to T-lymphocyte activation [36]. Additionally, we observed a concomitant enhanced CD62L expression, which is indicative of reduced activation [37]. The failure of *PI3Kδ−/−* cells to down-regulate CD62L has also been observed by others, who documented the importance of CD62L as regulator of lymphocyte recirculation [38]. Whereas defective T cell recirculation may *e.g.* explain the increased susceptibility of *PI3Kδ−/−* mice towards *Leishmania major* second infection [39], it is unlikely that it accounts for the increased tumor growth we observed. We conclude that several deficiencies contribute to impaired immunosurveillance by PI3Kδ-deficient CTLs: (i) *In vivo*, *PI3Kδ−/−* CTLs acquire a phenotype reminiscent of naïve cytotoxic T cells. In contrast to terminally differentiated effector CTLs (CD62L^low^, full equipment with lytic machinery), naïve cells (CD62L^high^) are only partially equipped and do not function properly. Indeed, the cytolytic capacity of *PI3Kδ*-deficient T cells was found to be severely impaired both *in vivo* and *in vitro*. (ii) An additional factor leading to this naïve-like phenotype is the inability of *PI3Kδ−/−* CD8+ T cells to react to foreign antigens *via* clonal proliferation. This was substantiated by the results obtained in the mixed lymphocyte reaction. (iii) Impaired secretion of IFN-γ by *PI3Kδ−/−* CTLs is likely to contribute to defective tumor surveillance [40], given the observation that PI3Kδ regulates IFN-γ production in several lymphocytic lineages [26], [28] and is required for TCR-induced IFN-γ production [39], [41].

The therapeutic concept of PI3Kδ inhibition in the treatment of hematopoietic malignancies is based on the knowledge that the PI3Kδ isoform is important in sustaining growth of leukemic cells [4], [16], [26], [42]. Accordingly, the specific PI3Kδ inhibitor CAL-101 have already entered clinical trials for the treatment of hematopoietic malignancies [17], [18], [43], [44]. Similarly, inhibitors for AKT are under development [45]. However, AKT is a questionable target. Due to its wide expression pattern, its inhibition is predicted to affect many cellular functions. Isoform-specific inhibitors are not yet available and their applicability is considered doubtfully [46]. At the current stage, specific PI3Kδ-inhibitors can be assumed to be superior to AKT-inhibitors. We believe that these compounds will have fewer side effects (due to restricted expression pattern of PI3Kδ) and be more efficacious.

Nevertheless, PI3Kδ-specific inhibition has unintended consequences on the functional activity of CTLs at all levels: activation, antigen-induced clonal expansion, degranulation and cytotoxicity. Therefore, unintended side effects such as impaired CTL-mediated tumor surveillance might counteract or even reverse intended treatment effects of PI3Kδ-inhibitors. As a consequence, in the clinical development of PI3Kδ-inhibitors, rigorous monitoring of tumor development should be implemented. Additionally, contraindications should be carefully defined, especially with respect to pre-existing immunodeficiency and/or tumor development in a patients history. However, while impaired CTL-function may be a drawback in cancer therapy, it is evident that the suppression of CTLs would be of profound benefit in the treatment of autoimmune diseases and other CD8+ T cell-associated diseases, such as COPD (chronic obstructive pulmonary disease) [47] and even more, in transplantation medicine. This concept is supported by a recent study of Ying et al [48], who investigated the therapeutical potential of pharmacological PI3Kδ inactivation in murine models of heart and skin transplantations.

## Materials and Methods

### Ethics Statement

All experiments were conducted in accordance with protocols approved by the Animal Welfare Committee of the Medical University of Vienna (66.009/0155-II/3b/2011) and the Austrian Federal Ministry of Science and Research.

### Mice and Cell Lines

Wild type (WT) C57BL/6 and BALB/c mice were purchased from Charles River Laboratories. *PI3Kδ−/−* animals [5] were backcrossed to C57BL/6 background. OT-1 mice were a kind gift from A.M. Dohnal (St. Anna Children’s Cancer Research Institute, Vienna, Austria). All animals were housed under pathogen-free conditions according to recommendations of the European Laboratory Animal Science Associations. All experiments were done with gender- and age-matched 8- to 12-week old animals. The murine colon adenocarcinoma cell line MC38 (kindly provided by V. Sexl, University of Veterinary Medicine, Vienna, Austria [23]), the murine thymoma cell line EL4 and OVA-expressing EG7 cell line (a kind gift from D. Stoiber-Sakaguchi, Ludwig Boltzmann Institute for Cancer Research, Vienna, Austria [49]) were propagated in Dulbeccós Modified Eagle Medium (DMEM, PAA) high glucose supplemented with 10% heat-inactivated fetal calf serum (FCS, PAA), 50 µM 2-mercaptoethanol (Sigma), 100 U/ml penicillin and 100 µg/ml streptomycin (PAA).

### Pharmacological Inhibition of PI3Kδ

The selective PI3Kδ inhibitor CAL-101 was purchased from Selleck Chemicals, IC-87114 was provided by ICOS (Bothell, WA). Inhibitors were used at the indicated concentrations. DMSO (ROTH) treatment served as negative control.

### Generation of Splenocytes and Expansion of aCD3-activated T Cells in vitro

Spleens from WT and *PI3Kδ−/−* animals were collected and forced through a 70 µm cell strainer. The resulting single cell suspension was treated with red blood cell lysis buffer. T cells were activated *in vitro* by stimulation with aCD3ε antibody (clone 145-2C11; 0.5 µg/µl) and expanded in culture for 3 days in T cell medium composed of RPMI-1640 containing L-glutamine (PAA) and supplemented with 10% FCS, 50 µM 2-mercaptoethanol, 1 mM sodium pyruvate (Gibco), non-essential amino acids (PAA), 100 U/ml penicillin, 100 µg/ml streptomycin and 100 U/ml rhIL-2 (Proleukin®, Novartis).

### Mixed Lymphocyte Reaction (MLR)

BALB/c splenocytes were growth arrested by treatment with mitomycin C (50 µg/ml, 20 min, Sigma). C57BL/6 WT and *PI3Kδ−/−* splenocytes were labeled with 2.5 µM carboxyfluorescein succinimidyl ester (CFSE). 1×10^5^ CFSE+ responding splenocytes (C57BL/6) were co-cultured in a ratio of 1∶1 and 1∶4 with stimulating splenocytes (BALB/c). Pharmacological inhibition of PI3Kδ was achieved by treatment of C57BL/6 WT splenocytes with 0.1 µM, 0.5 µM and 1 µM CAL-101 during the entire experimental procedure. After 24 h, 36 h, 48 h, 60 h, 72 h and 84 h co-cultures and controls were harvested and stained with aCD8-APC to determine CD8+-specific T cell proliferation by flow cytometry. Dead cells were excluded from analysis via gating in the FSC/SSC dot plot. % proliferating CD8+ cells define the percentage of cytotoxic T cells showing reduced CFSE intensity compared to the initial CFSE staining intensity.

### CFSE Proliferation Assay

Splenocytes were labeled with 2.5 µM CFSE and cultured in T cell medium supplemented with 0.5 µg/µl aCD3ε antibody (clone 145-2C11) at a concentration of 5×10^5^ cells/ml. After 12 h, 48 h, 62 h and 86 h splenocytes were harvested and stained with aCD8-APC antibody to determine CD8+-specific T cell proliferation characterized by the incremental loss of CFSE intensity in a flow cytometer. Dead cells were excluded from analysis via gating in the FSC/SSC dot plot.

### [^3^H]-thymidine Incorporation Proliferation Assay

aCD3-stimulated T cells were plated at a density of 5×10^4^/96 round-bottom well in triplicates in the presence of 0.1µCi/well [^3^H]-thymidine (PerkinElmer) for 48 h. Incorporated radioactivity was analyzed by adding Rotiszint® eco plus (ROTH) and measured in a liquid scintillation analyzer (Tri-Carb 1900 CA, PerkinElmer, Waltham, MA).

### Ca^2+^ Measurement

1×10^6^ splenocytes were stained with 1 µM Indo-1 AM (Invitrogen) in 100 µl cell loading buffer (Hanks Buffered Salt Solution (HBSS, Invitrogen) containing 1 mM Ca^2+^, 1 mM Mg^2+^ and 1% (v/v) FCS) for 30 min at 37°C and 5% CO_2_. After washing twice, cells were again incubated for 30 min at 37°C in cell loading buffer. Indo-1 AM loaded cells were stained for CD8 (clone 53-6.7). After staining, cells were resuspended in cell loading buffer. Cells were gated for CD8 expression and analyzed at 37°C on a LSRII flow cytometer (BD Bioscience, Heidelberg, DE). Ca^2+^ flux was either induced by using 1 µM thapsigargin (Sigma) or by stimulation with a biotinylated aCD3 antibody (10 µg/ml) followed by crosslinking with streptavidin (Sigma) (10 µg/ml). 3.3 µM ionomycin (Sigma) was used to induce full-scale deflection of internal Ca^2+^. The median fluorescence intensity of the Indo-1 AM violet versus Indo-1 blue ratio, obtained using FlowJo software (Tree Star), of at least three samples were combined using GraphPad Prism® and plotted against time.

### Quantitative Real-time PCR

1×10^6^ *in vitro* expanded aCD3-activated splenocytes were analyzed under basal conditions and after stimulation with 5 ng/ml IL-12 (R&D Systems) for 4 h. Total RNA was purified using Trifast reagent (Peqlab) followed by alcohol precipitation. 1 µg of RNA was reversely transcribed using the iSCRIPT cDNA synthesis kit (Biorad). qRT-PCR was performed on an Eppendorf Mastercycler® *realplex^2^* (Hamburg, DE) with SsoFast™ EvaGreen®Supermix (Biorad). Glycerinealdehyd-3-phosphat-dehydrogenase (*Gapdh)* was used for normalization. Following primers were used: *gapdh* forward: 5′-TGTGTCCGTCGTGGATCTGA-3′ and *gapdh* reverse: 5′-CCTGCTTCACCACCTTCTTGA-3′; *ifng* forward: 5′-AAGTGGCATAGATGTGGAAG-3′ and *ifng* reverse: 5′-GAATGCATCCTTTTTCGCCT-3′; granzyme A *(grzmA)* forward: 5′-TTCATTCTTGGGGCTCACTC-3′ and *grzmA* reverse: 5′-GGCATCTGGTTCCTGGTTTC-3′; granzyme B *(grzmB)* forward: 5′-CCAATCAGATATGTGCGGG-3′ and *grzmB* reverse: 5′- GGCTGCAGGCATAGTTTCC-3′; perforin 1 *(prf1)* forward: 5′-GATGTGAACCCTAGGCCAGA-3′ and *prf1* reverse: 5′-CCTGCTTCACCACCTTCTTGA-3′. Primers for PI3K p110α (*pik3ca*), p110β (*pik3cb*), p110γ (*pik3cg*) and p110δ (*pik3cd*) are described in [50].

### IFN-γ ELISA

1×10^6^ splenocytes were cultured in 96 round bottom wells with or without concanavalin A (ConA, Sigma) (10 µg/ml) for 48 h. IFN-γ release was measured using Quantakine Mouse IFN-γ ELISA according to the manufacturer’s protocol (R&D Systems).

### Capacitance Measurements

Capacitance measurements were performed in the whole-cell mode of the patch-clamp technique using the time domain method of Lindau and Neher [51]. As described for NK cells [26], as soon as stable whole-cell conditions were established, recordings of aCD3-stimulated and *in vitro* expanded T cells were started under superfusion (DAD-8-VC superfusion system) with control solution (140 mM NaCl, 2.5 mM KCl, 1 mM CaCl_2_, 1 mM MgCl_2_, 10 mM HEPES NaOH, pH 7.4) with a sample rate of 330 kHz and continued under superfusion with Ca^2+^-ionophore (1 µM ionomycin dissolved in DMSO) and 500 nM phorbol myristyl acetate (PMA, Sigma). IC-87114 was used at 1 and 4 µM concentrations added to standard medium 1 hour prior to the analysis; DMSO treatment served as control. Cell capacitance was calculated by integrating the area under the capacitive current transiently elicited by a 20 ms voltage step from −120 to −80 mV. This area was divided by the applied change in voltage (40 mV). Capacitance-measurements were performed at room temperature (22±1.5°C) using an Axoclamp 200B patch clamp amplifier (Axon Instruments, Foster City, CA). Pipettes were pulled from aluminum silicate glass (AF150-100-10, Science Products, Hofheim, Germany) with a P-97 horizontal puller (Sutter Instruments, Novato, CA), heat-polished on a microforge (MF-830, Narishige, Japan), and had resistances between 1 and 2 MΩ when filled with the recording pipette solution (105 mM CsF, 10 mM NaCl, 10 mM EGTA, 10 mM HEPES, pH 7.3). Voltage-clamp protocols and data acquisition were performed with pclamp 6.0 software (Axon Instruments) through a 12-bit A-D/D-A interface (Digidata 1200; Axon Instruments).

### Flow Cytometric-based Degranulation Assay

Degranulation of cytotoxic cells was quantified by monitoring the expression of CD107a on the cell surface as described previously [20]. Degranulation of WT and *PI3Kδ−/−* CTLs was triggered by 100 nM PMA and 0.33 µM ionomycin, or due to chronic low-frequency electrical stimulation. The PI3Kδ inhibitor CAL-101 was applied at different concentrations (0.1 µM, 0.5 µM, 1µ M and 5 µM) two hours prior to the stimulation with PMA/iono.

### Chronic Low-frequency Electrical Stimulation (CLFES)

WT and *PI3Kδ−/−* aCD3-activated CTLs were seeded at a cellular density of 3×10^5^ cells/ml T cell medium. Electrical stimulation was performed using the C-Pace EP from IonOptix Corporation (Milton, USA). Stimulation pulses of 5V amplitude and 5 ms duration were delivered *via* two carbon electrodes to 3.5cm dishes at a frequency of 1Hz. Three stimulation time periods (30 min, 10 min and 1 min) were tested. After the stimulation CTLs were directly subjected to the FACS-based analysis: stimulation-induced surface expression of CD107a was compared to the unstimulated situation (see above, flow cytometric-based degranulation assay). The most pronounced degranulation was obtained if CTLs were subjected to continuous stimulation for 10 minutes.

### In vivo CTL Assay

*In vivo* cytotoxicity was measured according to Schellack et al [52]. In brief, mice were immunized by subcutaneous injection of 0.1mg/mouse SIINFEKL (Bachem) in combination with the adjuvant CpG-ODN 1668 (Eurofins). Seven days later control mice and immunized mice received syngeneic splenocytes labeled with three different concentrations of the intracellular dye CFSE: 2.5, 0.25 and 0.025 µM. The CFSE^high^ population was pulsed with the relevant SIINFEKL peptide (10 µg/ml), the CFSE^mid^ population was pulsed with the irrelevant peptide m-TRP2_181–188_ (10 µg/ml, Bachem) and the CFSE^low^ population remained untreated. The three CFSE+ populations were mixed in a 1∶1∶1 ratio and 3×10^7^ cells were injected via the tail veins of recipient WT and *PI3Kδ−/−* mice. After 18 hours spleens and draining lymph nodes were removed. Single cell suspensions were analyzed by flow cytometry. Specific killing was calculated as [1-(% CFSE^high^/% CFSE^low^)] x 100.

### Generation of Peptide-reactive T Cells

Mice were immunized by subcutaneous injection of the OVA agonist peptide SIINFEKL (0.1 mg/mouse) in combination with the adjuvant CpG-ODN 1668. After seven days, mice were boosted by a second injection. Spleens were removed at day 14 and suspensions of splenocytes were obtained from control mice and immunized mice as described above. In parallel, splenocytes from WT mice were prepared, irradiated (30 Gy – gamma ray) and pulsed with SIINFEKL (10 µg/ml in RPMI-1640 medium containing L-glutamine and supplemented with 10% FCS, 50 µM 2-mercaptoethanol, 100 U/ml penicillin, and 100 µg/ml streptomycin). 5×10^6^ splenocytes of immunized or control mice were co-cultured with 2×10^6^ irradiated, SIINFEKL-pulsed splenocytes in 1 ml T cell medium for 5 days.

### In vitro Cytotoxicity Assay

Splenocytes of control mice and splenocyte suspensions containing peptide-reactive T cells were co-cultured with 5×10^4^ CFSE-stained (2.5 µM) EL4 or EG7 target cells at effector-to-target (E:T) ratios of 30∶1, 15∶1, 5∶1 and 1∶1 in triplicates in 96-well plates. In parallel, tumor cells were incubated in the absence of splenocytes to assess the extent of spontaneously occurring apoptosis. After 18 h, 5×10^4^ PKH26-stained control cells were added to each well as internal control and cytotoxicity was quantified *via* flow cytometry. 10^4^ labeled cells (either CFSE+ or PKH26+) were counted and the CFSE+ target cells were calculated as percentage of labeled tumor cells. Dead target cells were discriminated from living cells in a control staining with propidium iodide (Sigma) and further distinguished by determination of forward and sideward scattering. The percentage of specific lysis was calculated as [1– (%CFSE+ target cells after co-incubation)/(%CFSE+ cells without co-incubation)]×100.

### MC38 Tumor Model and Analysis of Infiltrating Lymphocytes

The flanks of WT and *PI3Kδ−/−* animals were depilated and 1×10^6^ MC38 cells were injected subcutaneously in each flank. After 15 days tumors were removed and weighed. For flow cytometric analysis the tumors were minced and digested with 1 mg/ml collagenase D (Roche Applied Sciences) and 0.05 mg/ml DNase I (Roche Applied Sciences) for 1h at 37°C. The digested tissue suspension was squeezed through a 70 µm cell strainer and washed twice with PBS prior to antibody staining.

### Antibodies and Flow Cytometric Analysis

The following antibodies were purchased from eBioscience: aCD3ε-APC, aCD8α-Alexa Fluor 488, aCD44-PE, aCD45RB-biotin, aCD62L-APC-eFluor 780, Streptavidin-PerCP-Cy5.5 and aCD16/CD32 as Fc receptor block. The antibodies aCD3ε-PerCP-Cy5.5, aCD8α-PE, aCD69-PE-Cy7 and aCD107a-FITC were ordered from BD Biosciences. The following cell dyes were used according to the manufacturer’s protocol: Cell Trace CFSE Cell Proliferation Kit (Invitrogen) and PKH26L Red Fluorescent Cell Linker Kit (Sigma). Samples were analyzed using a FACSCanto™ II flow cytometer (BD Biosciences, Heidelberg, DE) and analyzed with FACSDiva software version 6.1.2 (BD Biosciences) or with FlowJo software version 7.6.1 (Tree Star).

### Cell Cycle Analysis

Cell cycle and apoptosis analysis were conducted according to Hoelbl et al [53]. Dead cells were defined as cells with DNA contents lower than 2 n (sub-G0/G1).

### Whole Cell Extracts and Western Blot Analysis

Cells were lysed in 50 mM Tris/HCl pH 8.0, 10% (v/v) glycerol, 25 mM EDTA, 150 mM NaCl (all from ROTH), 2 mM DTT, 0.5% NP40 (Igepal CA-630), 25 mM NaF, 1 mM sodium vanadate, 0.5 mM PMSF, SIGMA*FAST* Protease Inhibitor (all from Sigma Aldrich Austria), and cell debris removed by centrifugation and re-suspended in 50 µl 2× Laemmli sample buffer. Proteins were separated with SDS-PAGE and blotted onto nitrocellulose membranes (Hybond, GE Healthcare Austria). PageRuler® Prestained Protein Ladder (Fermentas ThermoScientific Austria) was used as molecular weight standard. Membranes were probed and analyzed using the ECL Western blotting detection system (GE Healthcare Austria). The antibodies PhosphoDetect™ aAKT1-pThr^308^ (Calbiochem) and aAKT (Cell Signaling Technology, New England Biolabs GmbH Germany) were a kind gift from J. Werzowa (Department of Clinical Pharmacology, Medical University of Vienna, Vienna, Austria). The peroxidase-conjugated secondary rabbit antibody was from GE Healthcare (Austria).

### Statistical Analysis

Student’s *t*-test, Mann-Whitney *U* test, paired *t*-test, One-sample *t*-test and One-Way ANOVA were performed using GraphPad Prism® version 5.00 for Windows, GraphPad Software, San Diego California USA, www.graphpad.com. Statistical analysis is indicated for each experiment specifically (*… p<0.05; **… p<0.01; ***… p<0.001).

## Supporting Information

Figure S1 **Expression of class I PI3K catalytic isoforms in** ***PI3Kδ−/−*** **CTLs.** WT and *PI3Kδ−/−* splenocytes were activated for 3 days with aCD3ε and cultured in T cell medium in order to obtain highly purified CTLs. mRNA expression of PI3Kα (*pik3ca*: WT: 0.0034±0.0006; versus *PI3Kδ−/−*: 0.0033±0.0005, n≥5, *p = 0.89*), PI3Kβ (*pik3cb*: WT: 33e-5±5,7e-5; versus *PI3Kδ−/−*: 37e-5±7,3e-5, n≥5, *p = 0.66*), PI3Kγ (*pik3cg*: WT: 0.015±0.0017; versus *PI3Kδ−/−*: 0.011±0.0008, n≥5, *p = 0.08*) and PI3Kδ (*pik3cd*: WT: 44e-5±11e-5; versus *PI3Kδ−/−*: not detected (n.d.), n≥5) was quantified via qRT-PCR and normalized to the house-keeping gene *gapdh*. Values represent means±SEM, unpaired *t*-test.
(TIF)

Figure S2 **Pharmacological properties of the selective PI3Kδ inhibitor CAL-101.** A. aCD3-activated C57BL/6 WT CTLs were cultivated in T cell medium supplied with varying concentrations of CAL-101, DMSO, or left untreated (w/o), respectively. Cell cycle analysis revealed that short-term (3 h, 6 h) treatment with CAL-101 did not affect CTL cell viability significantly (3 h: w/o: 41.5±2.9%; dmso: 42.4±3.6%; 0.1 µM: 41±0.7%; 0.5 µM: 40.5±4.2%; 1 µM: 40.5±4.6%; 5 µM: 41.3±4%; 6 h: w/o: 40.4±0.9%; dmso: 38.3±2.6%; 0.1 µM: 37±1.6%; 0.5 µM: 36.8±1.6%; 1 µM: 37.6±3%; 5 µM: 33.9±1.1%). B. Cell cycle profiles were comparable between CTLs without treatment (w/o: G0/G1: 76.9±1.7%, S: 15.5±1.3%, G2: 7.3±0.4%), DMSO (G0/G1: 78.6±1.1%, S: 14.6±1%, G2: 6.6±0.1%), 0.1 µM CAL-101 (G0/G1: 78.8±0.6%, S: 14.2±0.8%, G2: 6.6±0.2%), 0.5 µM CAL-101 (G0/G1: 77.6±0.5%, S: 15.1±0.5%, G2: 7.1±0%), 1 µM CAL-101 (G0/G1: 78.4±0.8%, S: 14.5±0.7%, G2: 6.8±0.1%) and 5 µM CAL-101 (G0/G1: 78.9±0.6%, S: 14.1±0.2%, G2: 6.8±0.4%), n = 2. Values represent means±SEM. One-Way ANOVA did not reveal any statistically significant differences. C. Western Blot analysis of cell lysates derived from aCD3-activated WT CTLs treated for 2 hours with indicated concentrations of CAL-101 or DMSO, respectively. Two different WT CTL preparations are depicted. P-AKT signals disappeared already upon PI3Kδ inhibition with 0.1 µM CAL-101. NFκB p65 served as loading control.
(TIF)

Figure S3 ***In vitro*** **cytotoxicity of WT OT-1 CTLs upon pharmacological inhibition of PI3Kδ.** To generate effector cells, splenocytes from an OT-1 mouse were isolated and co-cultured for 5 days with irradiated SIINFEKL-pulsed splenocytes. To determine peptide-reactive CTL cytotoxicity *in vitro*, CFSE-labeled OVA-expressing EG7 target cells were co-cultured with effectors in ratios of 15∶1, 5∶1 and 1∶1. Specific *in vitro* target cell killing was quantified by flow cytometry (E:T = 15∶1: WT: 22% specific lysis; versus *PI3Kδ−/−*: 9% specific lysis).
(TIF)

Figure S4 **CTL surface expression of CD44, CD62L and CD69 upon pharmacological inhibition of PI3Kδ.** *In vitro* assay on primary mature WT CTLs treated for three days with 0.5 µM or 1 µM CAL-101 or DMSO, respectively. Every 24 hours the expression levels of the activation and maturation markers CD44, CD62L and CD69 were measured on CD3+CD8+ CTLs via flow cytometry. Pharmacological inhibition of PI3Kδ gave rise to CTLs with reduced expression of CD44 and elevated levels of CD62L, while no differences were observed in the expression of CD69 compared to DMSO-treated WT controls. (Day 1: CD44: DMSO: 50.8±5.6, 0.5 µM: 32.6±2.6; 1 µM: 31.9±2.7; CD62L: DMSO: 13.1±0.8, 0.5 µM: 25.9±0.3, 1 µM: 27.5±1.1; CD69: DMSO: 10.9±0.3, 0.5 µM: 11.9±0.4, 1 µM: 12.1±0.3; n = 4, values represent mean fluorescent intensities±SEM, One-Way ANOVA and Tukey’s Post-Hoc Test).
(TIF)
