# Supplementary figures and images for: PI3Kδ Is Essential for Tumor Clearance Mediated by Cytotoxic T Lymphocytes
Source: PLoS One. 2012 Jul 13;7(7):e40852. doi: 10.1371/journal.pone.0040852 (PMC3396622; doi:10.1371/journal.pone.0040852)

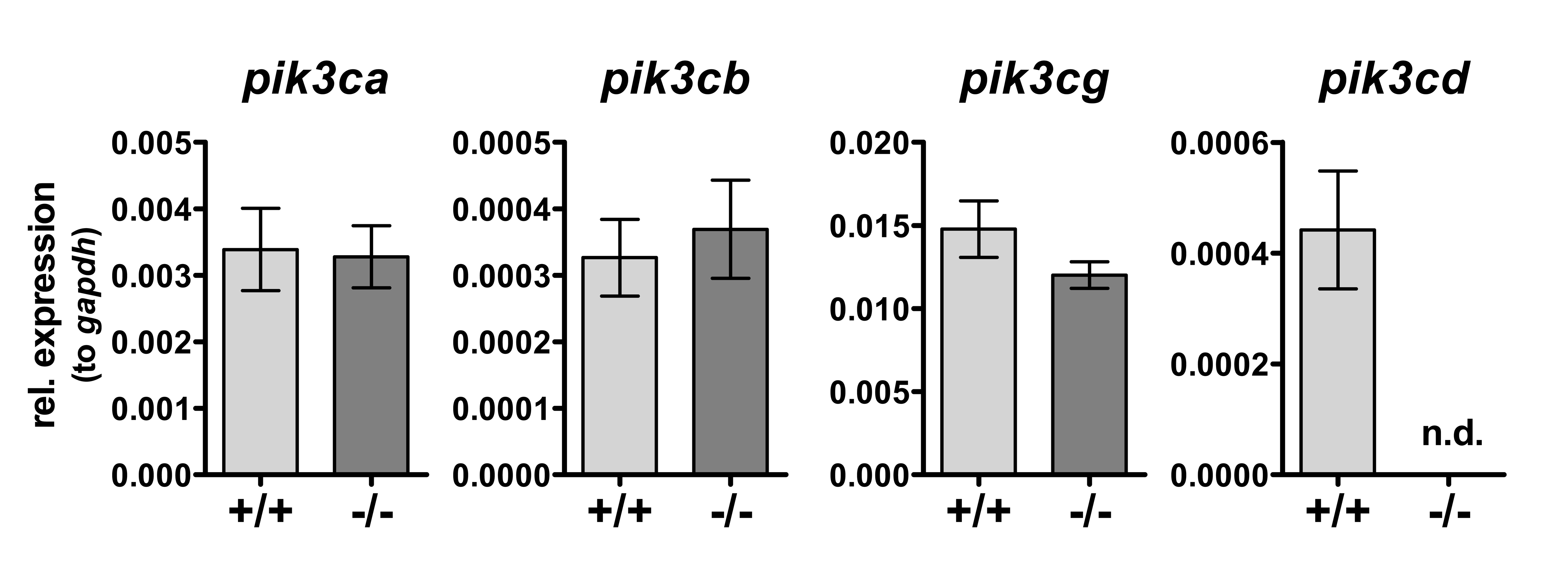

Supplement: Figure S1 — Expression of class I PI3K catalytic isoforms in PI3Kδ−/− CTLs. WT and PI3Kδ−/− splenocytes were activated for 3 days with aCD3ε and cultured in T cell medium in order to obtain highly purified CTLs. mRNA expression of PI3Kα (pik3ca: WT: 0.0034±0.0006; versus PI3Kδ−/−: 0.0033±0.0005, n≥5, p = 0.89), PI3Kβ (pik3cb: WT: 33e-5±5,7e-5; versus PI3Kδ−/−: 37e-5±7,3e-5, n≥5, p = 0.66), PI3Kγ (pik3cg: WT: 0.015±0.0017; versus PI3Kδ−/−: 0.011±0.0008, n≥5, p = 0.08) and PI3Kδ (pik3cd: WT: 44e-5±11e-5; versus PI3Kδ−/−: not detected (n.d.), n≥5) was quantified via qRT-PCR and normalized to the house-keeping gene gapdh. Values represent means±SEM, unpaired t-test. (TIF) [file pone.0040852.s001.tif]

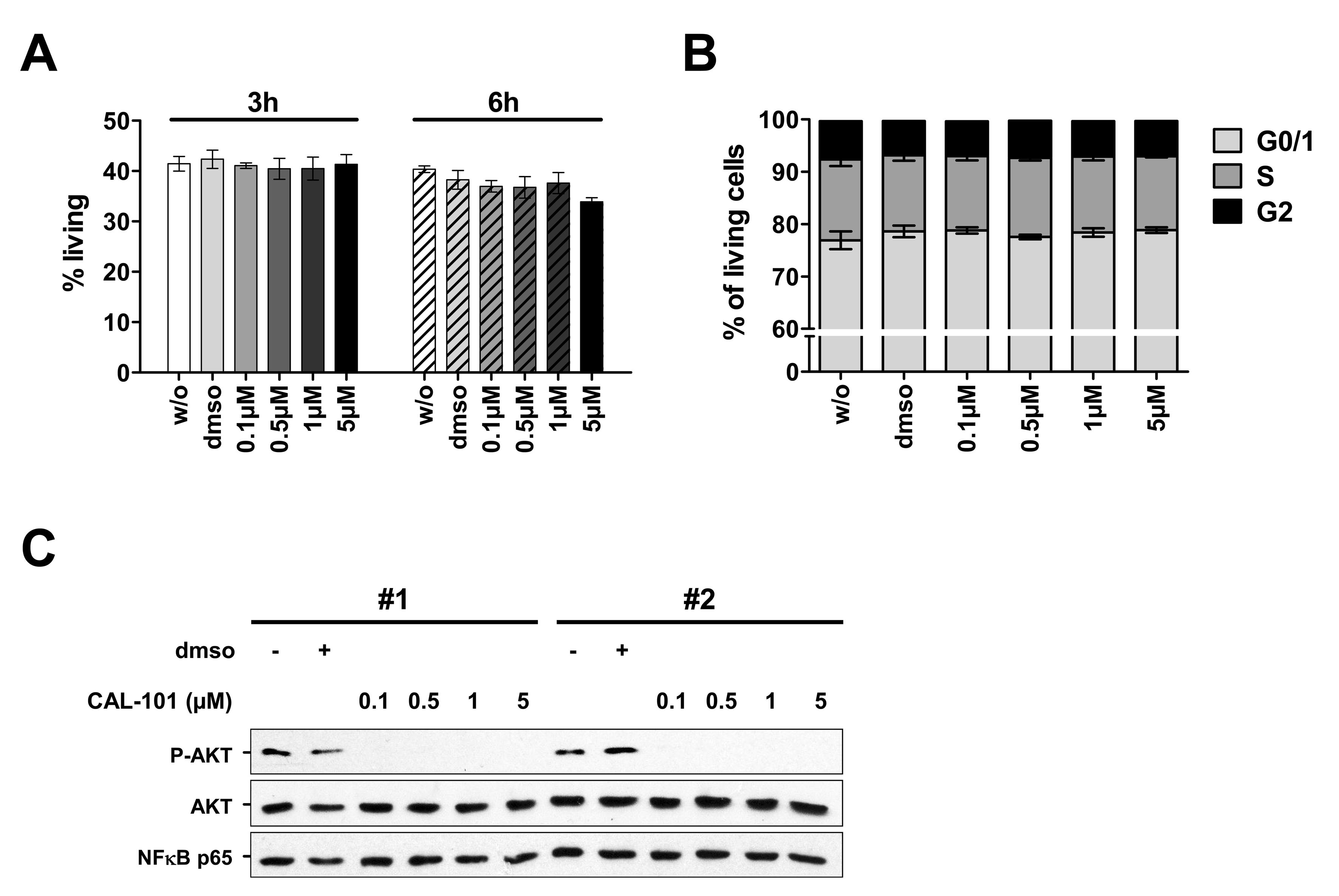

Supplement: Figure S2 — Pharmacological properties of the selective PI3Kδ inhibitor CAL-101. A. aCD3-activated C57BL/6 WT CTLs were cultivated in T cell medium supplied with varying concentrations of CAL-101, DMSO, or left untreated (w/o), respectively. Cell cycle analysis revealed that short-term (3 h, 6 h) treatment with CAL-101 did not affect CTL cell viability significantly (3 h: w/o: 41.5±2.9%; dmso: 42.4±3.6%; 0.1 µM: 41±0.7%; 0.5 µM: 40.5±4.2%; 1 µM: 40.5±4.6%; 5 µM: 41.3±4%; 6 h: w/o: 40.4±0.9%; dmso: 38.3±2.6%; 0.1 µM: 37±1.6%; 0.5 µM: 36.8±1.6%; 1 µM: 37.6±3%; 5 µM: 33.9±1.1%). B. Cell cycle profiles were comparable between CTLs without treatment (w/o: G0/G1: 76.9±1.7%, S: 15.5±1.3%, G2: 7.3±0.4%), DMSO (G0/G1: 78.6±1.1%, S: 14.6±1%, G2: 6.6±0.1%), 0.1 µM CAL-101 (G0/G1: 78.8±0.6%, S: 14.2±0.8%, G2: 6.6±0.2%), 0.5 µM CAL-101 (G0/G1: 77.6±0.5%, S: 15.1±0.5%, G2: 7.1±0%), 1 µM CAL-101 (G0/G1: 78.4±0.8%, S: 14.5±0.7%, G2: 6.8±0.1%) and 5 µM CAL-101 (G0/G1: 78.9±0.6%, S: 14.1±0.2%, G2: 6.8±0.4%), n = 2. Values represent means±SEM. One-Way ANOVA did not reveal any statistically significant differences. C. Western Blot analysis of cell lysates derived from aCD3-activated WT CTLs treated for 2 hours with indicated concentrations of CAL-101 or DMSO, respectively. Two different WT CTL preparations are depicted. P-AKT signals disappeared already upon PI3Kδ inhibition with 0.1 µM CAL-101. NFκB p65 served as loading control. (TIF) [file pone.0040852.s002.tif]

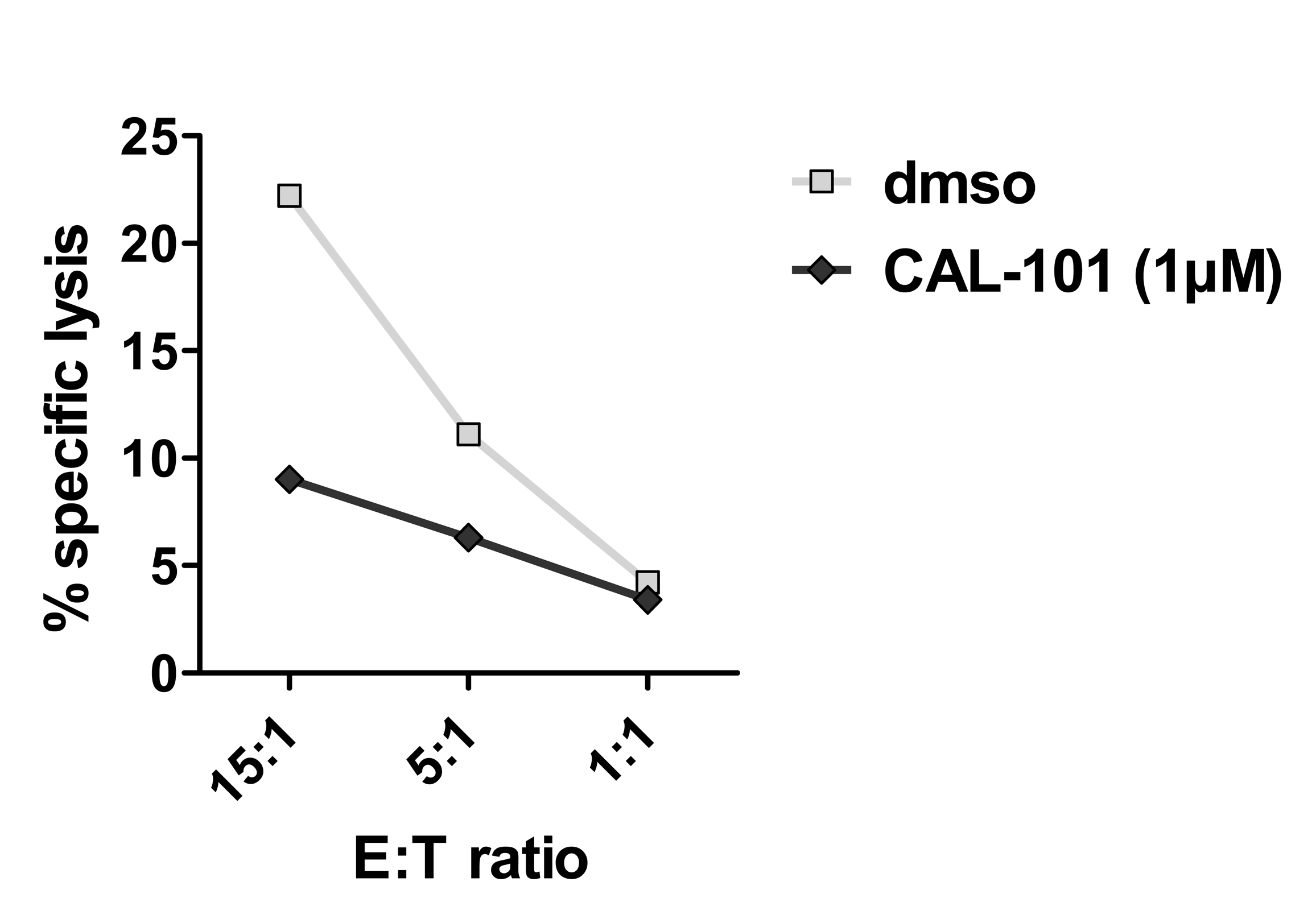

Supplement: Figure S3 — In vitro cytotoxicity of WT OT-1 CTLs upon pharmacological inhibition of PI3Kδ. To generate effector cells, splenocytes from an OT-1 mouse were isolated and co-cultured for 5 days with irradiated SIINFEKL-pulsed splenocytes. To determine peptide-reactive CTL cytotoxicity in vitro, CFSE-labeled OVA-expressing EG7 target cells were co-cultured with effectors in ratios of 15∶1, 5∶1 and 1∶1. Specific in vitro target cell killing was quantified by flow cytometry (E:T = 15∶1: WT: 22% specific lysis; versus PI3Kδ−/−: 9% specific lysis). (TIF) [file pone.0040852.s003.tif]

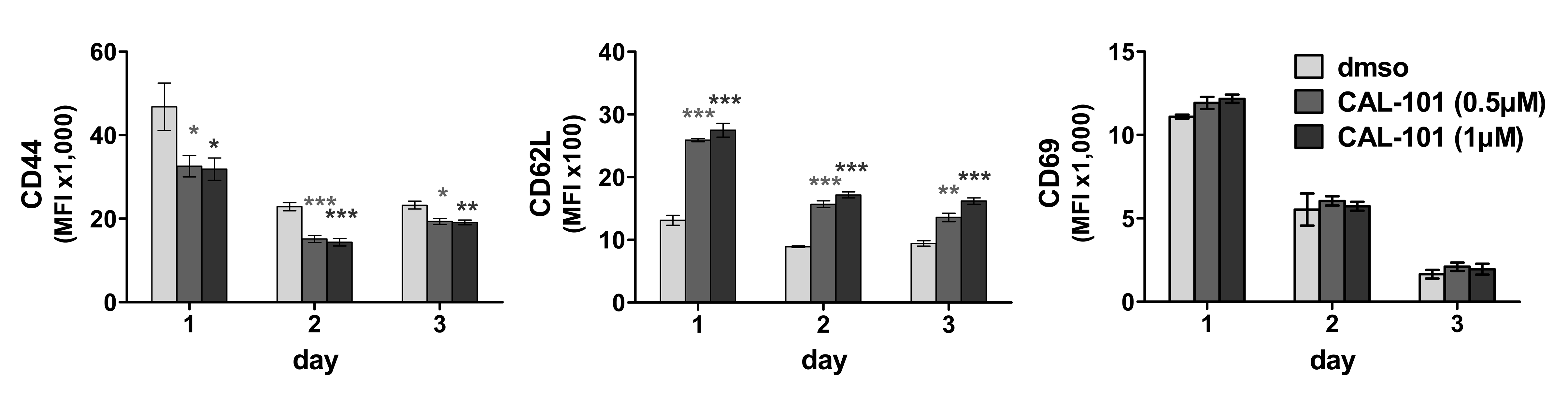

Supplement: Figure S4 — CTL surface expression of CD44, CD62L and CD69 upon pharmacological inhibition of PI3Kδ. In vitro assay on primary mature WT CTLs treated for three days with 0.5 µM or 1 µM CAL-101 or DMSO, respectively. Every 24 hours the expression levels of the activation and maturation markers CD44, CD62L and CD69 were measured on CD3+CD8+ CTLs via flow cytometry. Pharmacological inhibition of PI3Kδ gave rise to CTLs with reduced expression of CD44 and elevated levels of CD62L, while no differences were observed in the expression of CD69 compared to DMSO-treated WT controls. (Day 1: CD44: DMSO: 50.8±5.6, 0.5 µM: 32.6±2.6; 1 µM: 31.9±2.7; CD62L: DMSO: 13.1±0.8, 0.5 µM: 25.9±0.3, 1 µM: 27.5±1.1; CD69: DMSO: 10.9±0.3, 0.5 µM: 11.9±0.4, 1 µM: 12.1±0.3; n = 4, values represent mean fluorescent intensities±SEM, One-Way ANOVA and Tukey’s Post-Hoc Test). (TIF) [file pone.0040852.s004.tif]
